# The impact of humility traits on job search behavior in college graduates: the mediating role of job search clarity

**DOI:** 10.3389/fpsyg.2025.1602620

**Published:** 2025-07-11

**Authors:** Wen-Qian Zou, Si-Han Wang, Tian-Yi Xu, Qing-Wen Zheng

**Affiliations:** Child Research Institute, Ningbo Childhood Education College, Ningbo, China

**Keywords:** humility traits, job search clarity, job search behavior, college graduates, mediation analysis

## Abstract

**Introduction:**

This study investigates the influence of humility traits on job search behavior among college graduates, emphasizing the mediating role of job search clarity.

**Methods:**

A total of 406 college graduates were surveyed using the Humility Scale, the Job Search Clarity Scale, and the Job Search Behavior Scale.

**Results:**

The findings revealed that: (1) humility traits, job search clarity, and job search behavior are positively and significantly correlated; (2) humility traits significantly enhance job search clarity; (3) job search clarity significantly positively predicts job search behavior; and (4) job search clarity mediates the relationship between humility traits and job search behavior.

**Discussion:**

These results underscore the critical role of job search clarity in linking humility traits to proactive job search outcomes and offer valuable insights into supporting college graduates in their career development.

## 1 Introduction

In recent years, the number of college graduates in China has been steadily rising. According to the Ministry of Education, the number of graduates in 2024 is projected to reach 11.79 million. However, compared with this continuous growth in graduate numbers, the job market's capacity to absorb new entrants has not increased accordingly, and recruitment demand in some sectors has even declined.This imbalance between the growing number of graduates and labor market demands has intensified concerns regarding youth employment, drawing increasing attention to the challenges faced by college graduates. Given that job search behavior is a critical factor in employment outcomes—where a more proactive job search approach significantly enhances the likelihood of securing a job (Onyishi et al., 2015)—a deeper exploration of job search behaviors among college graduates is warranted. Understanding these behaviors is essential for addressing the broader challenges associated with graduate employment difficulties.

Individual traits play a crucial role as antecedents of job search behavior among college students (Bao and Luo, 2015; Boswell et al., 2012). For example, research has shown that a proactive personality significantly reduces career anxiety (Straud and McNaughton-Cassill, 2019) and positively influences job search outcomes (Brown et al., 2006). Humility, a trait highly valued in Chinese culture, has been extensively studied in organizational behavior, particularly in the context of humble leadership. Existing studies confirm that humble leadership fosters numerous positive outcomes for employees, including increased job satisfaction (Owens et al., 2013), improved job performance (Ou et al., 2014; Owens and Hekman, 2016), and enhanced wellbeing at work (Zhong et al., 2020). Recently, researchers in education have also begun to examine humility as an individual trait. Studies suggest that teacher humility enhances students' academic self-efficacy (Zou and Chen, 2024) and strengthens teacher-student relationships (Kwok et al., 2022). However, despite these findings, research on humility traits in educational settings remains limited. First, empirical studies on humility as an individual trait are scarce. Second, most existing research focuses on teachers, with relatively little attention given to students' humility traits. Third, studies on humility in education predominantly center on academic outcomes, neglecting other domains such as career development. These gaps highlight the need for further exploration of the potential benefits of humility traits among students.

**Figure 1:**
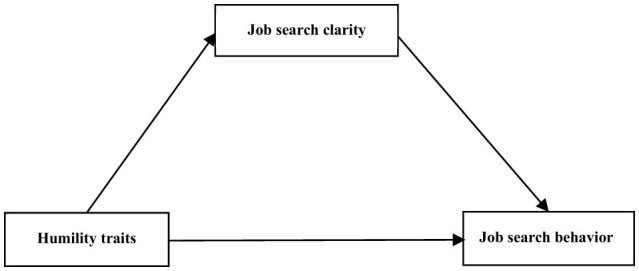
Theoretical model.

Scholars have underscored the practical significance of examining the role of humility traits in students' career development. For instance, Hou et al. (2019) found that humility traits positively influence students' future career identity by enhancing psychological availability, providing empirical support for career planning and intervention research. However, no studies have specifically investigated the relationship between humility traits and job search behavior among college students. Given that job search behavior is a key predictor of employment success (Onyishi et al., 2015), understanding the role of humility traits in shaping job search behavior holds substantial theoretical and practical value. To address this gap, the present study aims to examine the impact of humility traits on job search behavior among college graduates and explore potential mediating mechanisms. By doing so, this research seeks to expand the theoretical framework of job search antecedents and provide practical insights for improving career guidance in higher education institutions. This study makes the following contributions to the literature and existing theory. First, it extends humility research beyond organizational and leadership contexts to the domain of individual career development, with a particular focus on college graduates. Prior studies have primarily emphasized the effects of humble leadership on subordinates and teams (Owens et al., 2013; Owens and Hekman, 2016), while relatively less attention has been given to humility as an individual trait, especially among students. Second, the study situates humility traits within the framework of Social Cognitive Theory (SCT), empirically demonstrating their influence on job search behavior. This provides further support for the dynamic interaction between personal traits and behavioral outcomes as emphasized by SCT. Third, the study identifies job search clarity as a mediating mechanism linking humility traits to job search behavior. This finding not only advances existing literature on the role of cognitive clarity in career behavior but also reinforces the centrality of goal-setting in Social Cognitive Career Theory (SCCT), deepening our understanding of decision-making and behavioral processes in the job search context. The theoretical model developed for this study is presented in Figure 1.

## 2 Theory and hypotheses

### 2.1 Humility traits and job search clarity in college graduates

Humility is a multidimensional construct comprising three key components: acknowledging one's limitations, appreciating others' strengths, and teachability (Owens et al., 2013). Acknowledging one's limitations involves having an accurate self-assessment, recognizing both strengths and weaknesses, and proactively addressing shortcomings. Appreciating others' strengths refers to valuing the abilities of others without feeling threatened, while teachability reflects openness to external feedback and a willingness to learn. In the face of growing employment pressure, some college students experience blind self-doubt or overconfidence due to a lack of objective self-awareness, leading to vague job search goals and career indecision (Kang et al., 2022). Job search clarity, defined as the degree to which individuals have well-defined career goals and a structured job search plan, plays a crucial role in employment outcomes (Wanberg et al., 2002). Previous research has consistently highlighted that goal clarity is a foundational dimension of job search clarity, facilitating more structured and effective job search efforts (Côté et al., 2006; Stumpf et al., 1983; Wanberg et al., 2002). Students with humility traits are more likely to develop an objective self-assessment, allowing them to set realistic career goals and enhance job search clarity.

According to Social Cognitive Theory (Bandura, 1986, 1991), human functioning is shaped by the dynamic interplay among personal factors, behavior, and environmental influences—a principle known as triadic reciprocal determinism. Within this framework, the self-system plays a central role through mechanisms such as self-monitoring, self-evaluation, and self-regulation. These processes enable individuals to set goals, interpret feedback, and adjust their behaviors, thereby enhancing their goal-directedness and behavioral effectiveness (Bandura, 1991). Humility traits embody core elements of this self-system. Individuals high in humility tend to accurately appraise their abilities, accept external feedback, and revise their goals and actions through reflective self-regulation (Owens and Hekman, 2012). This ongoing adjustment process helps college graduates develop realistic, specific, and attainable career goals in the face of a complex job market.

Specifically, humble graduates are more likely to rationally assess their strengths and limitations, avoiding both undue self-doubt and overconfidence. By integrating accurate self-perception with external input, they clarify their developmental direction and formulate job search goals aligned with their competencies. Moreover, humility is closely associated with teachability—an openness to learning and receptiveness to guidance. This learning-oriented mindset enables individuals to better adapt to job market dynamics and identify industry trends, thereby enhancing the clarity of their job search objectives (Tangney, 2000). Prior research has also emphasized that accurate self-awareness and constructive use of feedback are essential for effective goal setting (Zimmerman, 2000; Côté et al., 2006). Based on this reasoning, the following hypothesis is proposed:

*Hypothesis 1:* Humility traits positively predict job search clarity among college graduates.

### 2.2 Job search clarity and job search behavior

Job search behavior refers to the proactive actions individuals take to seek employment opportunities, including the time and effort they invest (Blau, 1994). Prior research has shown that greater job search clarity enhances behavioral direction and efficiency. Students with high clarity are more likely to identify target positions quickly and engage in focused, sustained, and strategic job search activities. In contrast, individuals with vague goals may expend excessive effort exploring options, resulting in delayed and less effective actions (Wanberg et al., 2002).

Social cognitive theory highlights the critical role of goal setting in behavioral regulation (Bandura, 1989). Clear goals help individuals allocate cognitive resources more effectively, strengthen motivational regulation, and enhance the direction and persistence of behavior. Job search clarity, which entails individuals having well-defined employment goals and a structured plan, helps reduce cognitive conflict and the burden of decision-making, which in turn facilitates more persistent job search behavior. Moreover, specific and well-defined goals can enhance self-efficacy, thereby activating stronger motivational processes (Schunk and Zimmerman, 2006). This facilitates proactive behavior such as information gathering, adopting systematic job search strategies. Empirical research supports this mechanism. Earlier studies found that job search clarity significantly improves college students' preparedness and behavioral efficiency (Bao and Luo, 2015; Wang and Dai, 2017). More recent work further reveals that job search clarity not only directly predicts job search behavior, but also indirectly promotes sustained engagement by enhancing self-regulation and job search self-efficacy (Liu et al., 2022; van Hooft et al., 2021). Based on this reasoning, the following hypothesis is proposed:

*Hypothesis 2:* Job search clarity positively predicts job search behavior among college graduates.

### 2.3 The mediating role of job search clarity

Humble students are more likely to accurately recognize their strengths and limitations, enabling them to realistically evaluate the alignment between their capabilities and career aspirations. This self-awareness facilitates the setting of feasible and well-defined job search goals. In addition, they tend to actively acquire job search skills and seek guidance from experienced professionals, which helps clarify their career direction and enhances job search clarity. Students with clearly defined goals are better able to understand target positions and concentrate their attention on relevant job search activities. Therefore, humility traits can enhance job search clarity, which in turn promotes more proactive and goal-directed job search behavior.

According to social cognitive theory (Bandura, 1986, 1991), behavior, personal factors, and environmental influences interact dynamically to shape human functioning. The job market and employment context influence college students' cognition and behavior, while students can also adaptively regulate their cognition and actions in response to external changes. Building on this framework, Social Cognitive Career Theory (SCCT; Lent et al., 1994) and its career decision-making model offer a valuable theoretical lens through which to explore how personality traits influence career behavior. SCCT emphasizes that career goals are a core mechanism in career decision-making, and job search clarity is one of the most malleable cognitive variables in this process. Within the framework of this study, humility traits—as a type of personal factor—may affect behavioral outcomes (i.e., job search behavior) by shaping goal-related cognition (i.e., job search clarity). Specifically, humility may foster the development of clear and realistic job goals, thereby guiding individuals to engage in more focused, sustained, and purposeful job search behaviors.This mediating pathway is also supported by prior empirical findings. For example, Côté et al. (2006) identified job search clarity as a key mediating variable linking personality traits to job search intensity and employment outcomes, demonstrating its role in transmitting the influence of personal characteristics onto behavioral outcomes. Thus, this study presents the following hypothesis:

*Hypothesis 3*: Job search clarity mediates the relationship between humility traits and job search behavior among college graduates.

## 3 Method

### 3.1 Participants

A structured survey form was used for data collection. This study targeted final-year college students as the primary population. Given the need to complete data collection before students graduated and left campus, convenience sampling was adopted as a practical and efficient approach. As noted by Etikan et al. (2016), convenience sampling is an acceptable technique when the target population is clearly defined and data must be collected under time constraints. It has been widely employed in quantitative research within the fields of education and behavioral sciences.

The survey was administered via Questionnaire Star (https://www.wjx.cn), the largest online survey platform in China. Data were collected between January and February 2023 from graduating students at several universities in Zhejiang Province, China. With the assistance of university faculty members, the research team distributed the survey link and QR code through WeChat to student groups. All participants completed and submitted the questionnaire voluntarily and anonymously. The participants in this study represented diverse academic majors rather than being confined to a specific discipline (e.g., psychology). Such disciplinary diversity helps mitigate the risk of systematic bias stemming from field-specific influences on response patterns. Additionally, the survey instructions explicitly stated that participation was entirely voluntary and that respondents could withdraw at any time. Given the brevity of the questionnaire and the minimal burden placed on participants, no incentives were provided. As noted by Lavrakas (2008), “for simple and short mailed surveys, short phone interviews, and short Internet surveys, an incentive is not likely to be needed”.

A total of 505 questionnaires were distributed, and after excluding invalid responses, 406 valid questionnaires were retained, yielding a valid response rate of 80.39%. As shown in Table 1, among the valid respondents, 179 were male students (44.09%), and 227 were female students (55.91%). Regarding university type, 275 participants were from junior college (67.73%), while 131 were from 4-year university (32.27%). In terms of family residence, 103 participants were from rural areas (25.37%), 102 from townships (25.12%), and 201 from cities (49.51%).To evaluate the adequacy of the sample size, a sensitivity power analysis was conducted using G^*^Power 3.1 (Faul et al., 2007). This method estimates the minimum effect size that can be reliably detected based on a given sample size, significance level, and statistical power, thereby providing a basis for assessing the statistical sensitivity and robustness of the study (Prajapati et al., 2010). Based on a multiple regression model with a significance level of α = 0.05 and five predictors (including one independent variable, one mediator, and three control variables), the current sample size (*N* = 406) was sufficient to detect an effect size smaller than f^2^ = 0.01. This value is below the threshold for a small effect (f^2^ = 0.02) as defined by Cohen (1988), suggesting that the study has strong statistical sensitivity and is capable of detecting relatively weak effects, thereby enhancing the explanatory power and reliability of the findings.

**Table 1:** Basic information of the sample.

**Project**	**Types**	**Number of people**	**Proportion**	***N***
Gender	Male	179	44.09%	406
	Female	227	55.91%	
University type	Junior college	275	67.73%	406
	4-year university	131	32.27%	
Family residence	Rural areas	103	25.37%	406
	Townships	102	25.12%	
	Cities	201	49.51%	

### 3.2 Measures

The measurement instruments used in this study were all well-established scales published in reputable peer-reviewed journals, with demonstrated reliability and validity. As the original scales were developed in English, Brislin's (1986) translation–back translation procedure was employed to ensure semantic equivalence and cultural appropriateness in the Chinese version. All items were rated on a 7-point Likert scale (1 = strongly disagree, 7 = strongly agree).

#### 3.2.1 Humility traits

Humility traits were assessed using the 9-item humility scale developed by Owens et al. (2013). A sample item is: “I am willing to acknowledge my shortcomings and actively seek feedback, even when it is critical.” The Cronbach's alpha for this scale in the current study was 0.84.

#### 3.2.2 Job search clarity

Job search clarity was measured using the 5-item scale developed by Côté et al. (2006). A sample item is: “I have set a goal for the type of job I want to have when I graduate.” The Cronbach's alpha for this scale in the current study was 0.84.

#### 3.2.3 Job search behavior

Job search behavior was assessed using the 12-item scale developed by Blau (1994). A sample item is: “I spent a lot of time looking for a job alternative.” The Cronbach's alpha for this scale in the current study was 0.74.

#### 3.2.4 Control variables

Prior research suggests that demographic factors, including gender, university type, and family residence, may influence job search behavior among college graduates (Kanfer et al., 2001; Saks, 2006; Wang et al., 2017). Therefore, these variables were included as controls in the analyses.

### 3.3 Statistical analysis

The data collected in this study were analyzed using SPSS 26.0 and the SPSS macro program PROCESS. The data were analyzed using SPSS 26.0 and the SPSS PROCESS macro. The statistical analysis comprised three main steps: common method bias testing, preliminary statistical analysis, and hypothesis testing. First, exploratory factor analysis (EFA) was conducted to assess potential common method bias. Next, preliminary statistical analysis was performed in two steps: (1) descriptive statistics for humility traits, job search clarity, and job search behavior; (2) Pearson correlation analysis to examine relationships between variables. Hypothesis testing involved three steps: (1) hierarchical regression analysis to explore the predictive effect of humility traits on job search clarity (H1), after controlling for demographic variables; (2) hierarchical regression analysis to assess the predictive effect of job search clarity on job search behavior (H2), after controlling for demographic variables; (3) testing the mediating effect of job search clarity between humility traits and job search behavior using the Bootstrap method with the SPSS macro program PROCESS Model 4 (H3).

## 4 Results

### 4.1 Measurement model assessment

Confirmatory factor analysis (CFA) was conducted to examine the construct validity of the measurement model. This analysis assessed whether the items in the questionnaire appropriately loaded onto their respective latent variables and provided preliminary evidence of alignment between the theoretical model and the empirical data. The CFA model included three latent constructs: humility traits, job search clarity, and job search behavior. Results indicated that the factor loadings and standard errors for all items were statistically significant at the 0.05 level. The model demonstrated a good overall fit: χ^2^/df = 1.92, SRMR = 0.04, RMSEA = 0.05, GFI = 0.94, AGFI = 0.92, NFI = 0.91, TLI = 0.95, and CFI = 0.96. Following Fornell and Larcker (1981a), we assessed convergent validity using composite reliability (CR) and average variance extracted (AVE). As shown in Table 2, the CR values for all three constructs exceeded the recommended threshold of 0.70. The AVE for job search clarity was above 0.5, while the AVE values for humility traits and job search behavior were greater than 0.4. Although 0.5 is the commonly accepted cutoff for AVE, Fornell and Larcker (1981b) noted that when CR exceeds 0.6, an AVE value above 0.4 may still indicate acceptable convergent validity.To assess discriminant validity, we applied the Fornell–Larcker criterion by comparing the square root of each construct's AVE with its correlations with other constructs. As shown in Table 3, the square root of each AVE exceeded the corresponding inter-construct correlations, supporting adequate discriminant validity among all study variables.

**Table 2:** Result of Cronbach alpha, composite reliability and average variance extracted.

**Construct**	**Cronbach alpha**	**CR**	**AVE**
1. Humility traits	0.84	0.84	0.41
2. Job search clarity	0.84	0.84	0.51
3. Job search behavior	0.74	0.73	0.49

**Table 3:** Means, standard deviations, and correlations (*n* = 406).

**Variable**	***M***	***SD***	**1**	**2**	**3**	**4**	**5**	**6**
1. Gender	1.56	0.50						
2. University type	1.32	0.47	−0.03					
3. Family residence	2.24	0.83	0.03	0.02				
4. Humility traits	5.53	0.71	−0.06	0.12^*^	0.04	(0.64)		
5. Job search clarity	5.37	0.99	−0.09	0.07	−0.04	0.48^**^	(0.71)	
6. Job search behavior	5.33	0.92	−0.09	0.07	−0.01	0.48^**^	0.69^**^	(0.70)

1. Gender was coded 1 = male, 2 = female. 2. University type was coded 1 =Junior College, 2 = 4-year general university. 3. Family residence was coded 1 = rural area, 2 = township, 3 = city. 4. ^*^p < 0.05, ^**^p < 0.01. 5. Values in parentheses represent square roots of AVE.

### 4.2 Common method bias

To reduce the potential impact of common method variance (CMV), we adopted both procedural and statistical remedies as recommended by Podsakoff et al. (2003). First, participants were clearly informed in the survey instructions that their responses would remain anonymous and confidential, and would be used solely for academic purposes, in order to reduce evaluation apprehension and social desirability bias. Second, items measuring different constructs were presented in a randomized order to ensure psychological separation between predictors and outcomes, thereby minimizing consistency artifacts. Finally, Harman's single-factor test was conducted, following Podsakoff and Organ (1986), to statistically assess the presence of CMV. The results showed a KMO value of 0.93 and a Bartlett's test of sphericity value of 2,651.52 (*p* < 0.001). The first unrotated factor accounted for 36.40% of the variance. According to Podsakoff and Organ (1986), CMB is not a serious concern if a single factor explains <50% of the variance. Therefore, no significant CMB issue was detected in this study.

### 4.3 Descriptive statistics and correlational analysis

Prior to conducting the correlation analysis, we examined the normality of the data to ensure the appropriateness of applying parametric statistical techniques. Skewness and kurtosis are commonly used to evaluate whether data approximate a normal distribution, with values closer to zero indicating greater normality. According to the guidelines proposed by Kline (2015), data can be considered approximately normally distributed when the absolute value of skewness is below 3 and that of kurtosis is below 8. In this study, the skewness values for items measuring the three key variables ranged from −0.82 to −0.22, and the kurtosis values ranged from −0.54 to 1.38. These values fall within the recommended thresholds, indicating that the sample data meet the assumption of multivariate normality. Pearson correlation analysis was conducted, and the results are presented in Table 3. Humility traits were positively correlated with job search clarity (r = 0.48, *p* < 0.01), and job search clarity was positively correlated with job search behavior (r = 0.69, *p* < 0.01).

### 4.4 Hypothesis testing

To mitigate potential multicollinearity, we conducted a collinearity diagnostic using the Variance Inflation Factor (VIF) before hypothesis testing. VIF is a widely used indicator for assessing multicollinearity among predictors. Common guidelines suggest that VIF values below 10 indicate no serious multicollinearity, values between 10 and 100 suggest moderate multicollinearity, and values above 100 indicate severe multicollinearity (Hair et al., 2017). In this study, the VIF values for both the core explanatory variable and control variables ranged from 1.01 to 1.33, well below the threshold of concern. These results indicate that multicollinearity was not a significant issue, thereby supporting the reliability of the regression estimates.

Gender, university type, and family residence were included as control variables. Hierarchical regression analysis was conducted to test Hypothesis 1 and 2. The results indicated humility traits of college graduates significantly predicted job search clarity (β = 0.68, *p* < 0.001), supporting H1. Job search clarity significantly predicted job search behavior (β = 0.65, *p* < 0.001), supporting H2.

To test the mediation effect (H3), the SPSS PROCESS macro (Model 4) was applied, with bootstrapping based on 5,000 resamples (Shrout and Bolger, 2002). The results, shown in Table 4, indicate that the indirect effect of job search clarity between humility traits and job search behavior was 0.38. The 95% bias-corrected confidence interval was [0.29, 0.49], excluding zero, confirming the mediating role of job search clarity. Thus, H3 was supported. In addition, we calculated the Variance Accounted For (VAF) to further assess the significance of the mediation effect. VAF indicates the proportion of the total effect that is transmitted through the mediator variable. According to Hair et al. (2014), a VAF value below 20% suggests no mediation effect, a value between 20% and 80% indicates partial mediation, and a value above 80% reflects full mediation. In this study, the VAF value was 60.86%, indicating that job search clarity partially mediates the relationship between humility traits and job search behavior.

**Table 4:** Results of indirect effects.

**Variable**	**Job search clarity**		**Job search behavior**	
	**Model 1-1**	**CI (95%)**	**Model 1-2**	**CI (95%)**
Constant	1.91^***^ (0.40)	[1.13, 2.69]	0.95^**^ (0.30)	[0.36, 1.55]
Humility traits	0.68^***^ (0.06)	[0.56, 0.80]	0.24^***^ (0.05)	[0.14, 0.35]
Job search clarity			0.56^***^ (0.04)	[0.49, 0.63]
R^2^	0.24		0.52	
*F*	32.52^***^		85.51^***^	
Indirect effects	β = 0.38	SE = 0.05	CI (95%) = [0.29, 0.49]	

1. Values in parentheses represent standard errors. 2. ^**^p < 0.01, ^***^p < 0.001.

## 5 Discussion

### 5.1 The relationship between humility traits and job search clarity

This study identified a significant positive relationship between humility traits and job search clarity, humility traits positively predict job search clarity. This finding highlights the importance of humility in helping students set clear career goals, particularly in a competitive job market. Employment is a major livelihood issue, and as pointed out by the Minister of Education, Huai Jinpeng, during the 2022 press conference on “China's Decade”, the number of college graduates in China reached 10.76 million, marking the first time it surpassed 10 million. In this context, the employment challenges faced by college graduates have garnered increasing attention. During the job search process, some graduates struggle with unclear self-awareness, leading to blind confidence or self-doubt, which results in confusion and uncertainty (Kang et al., 2022). In contrast, students with humility traits are better able to objectively evaluate themselves, remain open to external information, and actively seek help from others (Kumar and Prieto-Flores, 2024; Ou et al., 2014). These traits help them clarify their job search goals and improve job search clarity. Understanding the factors influencing job search clarity is essential for guiding students in their career search (Wanberg et al., 2002). Research has long emphasized the role of individual traits in this process. For example, proactive personality has been shown to positively affect job search clarity (Shen and Hu, 2015; Zhu et al., 2021). Although some studies have examined the impact of humility traits on career development—such as Hou et al. (2019), who found that humility enhances college students' psychological availability and strengthens their future work self-clarity—no research has directly investigated the relationship between humility traits and job search behavior among college students.This study confirms that humility traits positively influence job search clarity among college graduates, further substantiating the role of individual traits in shaping job search behavior and contributing to the broader literature on the positive effects of humility.

### 5.2 The mediating role of job search clarity

This study found that job search clarity significantly and positively predicts job search behavior, supporting Hypothesis 2, which aligns with the findings of Bao and Luo (2015). Graduates with clearer job search goals are better able to avoid inefficient and aimless job search activities, allowing them to allocate their efforts more effectively within a limited timeframe (Locke, 2015). Furthermore, job search clarity was found to mediate the relationship between humility traits and job search behavior, supporting Hypothesis 3. This suggests that humility traits enhance job search behavior by improving job search clarity.This finding is consistent with the Social Cognitive Career Theory (Lent et al., 1994), which posits that goals are a core mechanism in career decision-making, and job search clarity is a key malleable factor in employment outcomes. Career decision-making difficulties (Miller and Rottinghaus, 2014) and ambiguity (Xu and Tracey, 2017) are major sources of anxiety for job seekers, and low job search clarity can exacerbate this anxiety and confusion. Previous research has shown that job search clarity is an important “bridge” influencing job search behavior. For example, proactive personality can indirectly improve job search outcomes by enhancing job search clarity (Brown et al., 2006) and reducing career anxiety (Straud and McNaughton-Cassill, 2019). Côté et al. (2006) found that job search clarity mediated relationships between positive affectivity and job search intensity. Ye et al. (2017) demonstrated that career calling can indirectly enhance employ ability by improving job search clarity. Building on these studies, this research confirms that humility traits positively influence job search behavior by enhancing job search clarity, further validating the importance of job search clarity as a key pathway in job search behavior.

## 6 Conclusion

Anchored in social cognitive theory, this study investigated the indirect effect of humility traits on college graduates' job search behavior through job search clarity. Using survey data from 406 college graduates in Zhejiang Province, China, we conducted regression and mediation analyses via SPSS and its PROCESS macro. The results revealed that humility traits significantly and positively predict job search clarity; job search clarity significantly and positively predicts job search behavior; furthermore, job search clarity mediates the relationship between humility traits and job search behavior.

## 7 Practical implications

From a practical standpoint, this study may have shed new light on career training programs targeted at college graduates. First, universities should actively cultivate humility traits in students by encouraging them to objectively evaluate their strengths and weaknesses, appreciate the abilities of others, and remain open to learning. Such traits not only enhance job search clarity but also provide a psychological foundation for long-term career development.

Second, in today's dynamic job market, many college students experience uncertainty and tend to follow employment trends without clear goals (Kang et al., 2022). Improving job search clarity is thus essential for guiding students toward effective employment strategies (Bao and Luo, 2015). To this end, efforts should be made at both institutional and individual levels. Institutions can support students by offering career planning courses, inviting industry experts and alumni to share experiences, and providing diverse career development resources (Yang et al., 2022). These actions help students establish clear career objectives and make more informed decisions. Meanwhile, students themselves should proactively engage in field-related internships or community-based programs to gain firsthand understanding of workplace expectations. Such experiential learning facilitates more targeted and strategic job search behaviors.

## 8 Theoretical implications

First, this study expands humility research beyond organizational and leadership domains into the realm of individual career development, with a particular focus on college graduates. Prior studies have predominantly examined the trickle-down effects of humble leadership, emphasizing how leaders' humility influences employees and teams (Owens et al., 2013; Owens and Hekman, 2016), while comparatively little attention has been given to the humble individuals themselves. In recent years, the education field has also begun to explore the role of humility; however, existing research has largely centered on the effects of teacher humility on students (Kwok et al., 2022; Zhang and Chi, 2025; Zou and Chen, 2024), with scant attention to how students' own humility traits shape their career development. By examining the influence of humility traits on job search behavior among college graduates, this study extends the limited research in the aforementioned areas and enriches the theoretical understanding of humility in the context of individual career development.

Second, this study investigates the influence of humility traits on job search behavior within the framework of Social Cognitive Theory (SCT). SCT posits that personal, behavioral, and environmental factors interact dynamically to shape human functioning (Bandura, 1986, 1991). Through empirical analysis, this study verifies the positive influence of humility traits on college graduates' job search behavior, thereby further supporting and extending SCT's propositions regarding the dynamic impact of personal traits on behavioral outcomes.

Third, this study highlights the importance of goal setting for individual behavior, as emphasized in Social Cognitive Theory. SCT suggests that clear and specific goals play a crucial role in guiding and regulating behavior (Bandura, 1989). The empirical findings of this study demonstrate that job search clarity mediates the relationship between humility traits and job search behavior. This finding not only corroborates and extends prior work identifying job search clarity as a key mediating variable between personality traits and job search outcomes (Côté et al., 2006; Ye et al., 2017), but also further elucidates the central role of career goals within the framework of Social Cognitive Career Theory (SCCT; Lent et al., 1994), thereby deepening the theoretical understanding of career decision-making and behavioral processes.

## 9 Limitations and directions for future research

This study has several limitations that warrant further consideration. First, as a cross-sectional design, it cannot establish causal inferences among the variables. Future studies should adopt experimental or longitudinal methods to more robustly validate the proposed causal pathways. Second, the sample was primarily drawn from universities in specific regions of China, and a convenience sampling method was employed, which may limit the generalizability of the findings. Broader and more diverse sampling across regions and institution types is recommended to enhance the external validity of future studies. Third, although anonymity and standardized instructions were employed to reduce social desirability bias, the reliance on self-reported measures may still introduce such bias. Future research could include a social desirability scale and control for it during statistical analysis (Umphress et al., 2010). Furthermore, exclusive reliance on self-report questionnaires constitutes another notable limitation. While these instruments provide valuable subjective insights, they fail to capture behavioral or physiological indicators that could offer a more comprehensive understanding of cognitive and emotional states. Future studies could consider integrating behavioral data such as facial expressions, gesture patterns, or multimodal indicators of agreement and disagreement. For instance, Buono et al. (2023) used facial behavior to assess student engagement in online learning, Poggi et al. (2011) explored multimodal expressions of agreement in debates, and Bousmalis et al. (2013) analyzed gesture patterns relevant to social interactions. Incorporating such observational or automated data would enrich analytical perspectives and provide a more nuanced depiction of user experiences and risk perceptions. Fourth, while job search clarity was examined as a mediator, other potential mediating mechanisms may also exist. Future studies could incorporate additional cognitive or emotional constructs, such as career decision-making self-efficacy or job search anxiety, to build a more comprehensive model and enrich understanding of the psychological processes driving job search behavior.Finally, given the increasing prevalence of online job searching among university students, future research should also explore potential risks inherent in this context, such as fraudulent job postings or uncertain scenarios, which represent significant real-world threats to students. Enhancing students' awareness and preparedness in identifying and managing these risks effectively is important. Future studies might examine strategies designed to improve risk perception and preventive behaviors through educational interventions or serious games. For example, D'Errico et al. (2022) demonstrated how serious games can effectively increase adolescents' awareness of health and security risks, highlighting a promising approach that could be adapted to the context of online job search.

## Data Availability

The raw data supporting the conclusions of this article will be made available by the authors, without undue reservation.
